# Evaluating the effectiveness of the SMART contract-signing strategy in reducing the growth of Swedish Adolescents’ substance use and problem behaviors

**DOI:** 10.1186/s12889-016-3131-9

**Published:** 2016-06-22

**Authors:** Cristian Bortes, Susanna Geidne, Charli Eriksson

**Affiliations:** School of Health and Medical Sciences Örebro University, SE-701 82 Örebro, Sweden

**Keywords:** Program evaluation, Prevention, Swedish, Adolescent substance use

## Abstract

**Background:**

In 2013, around 40 % of the schools in Sweden had structured programs to prevent tobacco and alcohol debut in compulsory school. There has unfortunately been a lack of scientific evidence to support most of the prevention methods focusing on primary prevention in schools in Sweden. The aim and purpose of the present study is to evaluate the effectiveness of the Non-Governmental Organization SMART contract-signing strategy in reducing the growth of youth substance use and other problem behaviors amongst Swedish adolescents.

**Methods:**

Students from five schools in a medium-sized Swedish municipality were surveyed in three waves from 7^th^ to 9^th^ grade of compulsory school. We used General Linear Model (GLM) repeated-measures ANOVA to test if the outcome measures smoking, use of snus and alcohol, drunkenness, delinquency, and bullying significantly changed different amounts over time in groups that had participated in the SMART program for long time, a short time, sporadically- or not at all. Groups were compared on demographic background variables, and outcome measures were assessed on all measurement occasions by a one-way ANOVA. The magnitude of group differences at the end of the study was estimated according to Cohen’s *d*.

**Results:**

Number of years with a contract has an effect on the levels of self-reported youth problems in 9^th^ grade. We found small to medium-sized differences in measured outcomes between students who participated in the program for the longest period of time, 5 years, and who participated for the shortest time, 0–2 years.

**Conclusion:**

Findings suggests that the SMART program has preventive effects on adolescent substance use.

## Background

Use of alcohol, tobacco and other illicit drugs usually begins during the adolescent years [1]. The associated risks are well known, as these problematic behaviors are among the most important causes of disease and mortality worldwide [2–4] and place an economic burden on society as well [5]. Early substance-use initiation in particular plays a significant role in later substance-related problems and is also related to psychosocial problems during young adulthood [6–8]. The risk of becoming nicotine dependent is greater for those who start smoking early in life than for those who start later [9], and hence the risk of becoming a regular smoker decreases as the onset age increases [10, 11]. Underage drinking and especially first use of alcohol between 11 and 14 years of age have been linked to a range of later health problems as well [12–16]. Preventing or delaying onset of youth substance use is therefore a great public health concern.

Youth substance use is a complex phenomenon, and it can be difficult to get an absolute and complete understanding of it or of how best to tackle it. We know that attitudes and norms [17], and characteristics of the social environment [18, 19] such as peer socialization [20], parental expectations [21] and smoking rules in school [22], as well as sociocultural influences [23, 24], are some of the determining factors, among others [25, 26], of smoking and alcohol use in adolescence. It is possible, however, based on knowledge of risk and protective factors, to reduce the extent to which youth problems develop into increasingly more harmful and long-term disorders [27]. There exist different approaches and methods to prevent youth problems, and as a primary preventive measure to address youth substance use, school-based interventions are common [28–37]. In Sweden, where schooling is compulsory, this provides the possibility to reach virtually all children.

In 2013, around 40 % of the schools in Sweden had structured programs to prevent tobacco and alcohol debut in compulsory school [38]. However, there has unfortunately been a lack of scientific evidence to support most of the primary prevention methods in schools in Sweden [39]. Hence, there is a need for evaluations in this national context, because without program evaluation resources can be wasted and misdirected. In particular, the concept of signing a contract to prevent tobacco and alcohol use has a long history in Sweden [40]. The non-governmental organization (NGO) SMART carries out one popular program in which positive reinforcement and signing of contracts with schoolchildren are core components. The purpose of this present study is to evaluate the effectiveness of their prevention program in reducing the growth of Swedish adolescents’ substance use and problem behaviors.

### Evidence from school-based contract-signing prevention programs

A variety of school-based programs for preventing use of alcohol, tobacco, and other drugs have been reported [28–37]. These comprise diverse types of interventions, populations, outcomes, and results. The conclusions that can be drawn are consequently limited to the specific contexts. The programs target determining factors known of smoking and alcohol use in adolescence. However, only a very few within this plethora of programs include contract signing as a component.

One well-studied school-based prevention program containing components found to be effective as well as the element of contract signing is the “Smoke-Free Class Competition” (SFC) [41]. It has the objective to delay or prevent the onset of smoking during adolescence. The SFC competition is considered to have amassed a rather broad body of evidence for its effectiveness as a school-based prevention program [41–44] and it has been widely implemented throughout Europe, though not in Sweden.

A tobacco prevention program that is well established and widely disseminated in Sweden is the “Tobacco-Free Duo” [45], which includes contract signing among other components. Despite its proven sustainability within communities, however, Tobacco-Free Duo still fails to fulfill the standards of evidence and the criteria of effectiveness as postulated by the Society for Prevention Research [46], since only one evaluation study [45], as part of a dissertation [47], has been conducted and published. We argue that there is an undeniable need to broaden the evidence-base for school-based preventive methods in Sweden, particularly those with a strategy of contract signing, as these are widely used but lack sufficient evidence for their effectiveness.

### The Non-governmental organization SMART and the contract strategy

Founded in 2001, SMART is a network for anyone involved in drug prevention. Its aim is to prevent or delay the onset of alcohol, tobacco and illicit drug use among schoolchildren through positive reinforcement and the signing of contracts. SMART’s method, the contract strategy, is based on voluntary participation and encourages young people to consciously opt out of unwanted behaviors and to make “smart” choices. SMART uses a whole-community approach, where local actors, with the support of SMART, design the method based on local conditions. More than 30,000 young people in Sweden are locally connected to some form of contract activity [40]. Today SMART is found in approximately 90 Swedish municipalities, as well as in 8 municipalities in other countries. Actors behind these contract activities may be county councils, social services, police, schools, sports clubs and NGOs. Having different actors heading the contract activities in different locations also means that there are differences in the implementation and delivery of the program from one place to another. The relative levels of emphasis on fun activities, as reinforcement for the students, or on financial incentives, such as discounts and lotteries, vary between actors. While the program in general targets 10–16 year-olds in compulsory school, decisions on what school-grades to target can differ. There are also many different names for the local operations. The membership cards are different, and some operations do not have membership cards. SMART represents a general concept, an overall strategy that is adapted to local conditions, rather than necessarily being a uniform, manual-based, step-by-step program. The major exception is Tobacco-Free Duo, the largest variant within the network SMART, which works with a clearly mapped manual. Despite local differences, a minimum requirement is that the contract must contain an agreement concerning tobacco use. The idea is that students sign a contract at the beginning of the school year, and a parent must give written consent. The contract is an agreement whereby the student promises to refrain from smoking cigarettes, using snus (Swedish moist snuff), or using other tobacco products during the coming year. The contract may contain additional items as well; for instance the program-version we evaluate in this study includes abstaining from using drugs (such as tobacco, alcohol, drugs, sniffing agents and dopants), destroying other people’s belongings, shoplifting, or stealing, and also includes being a good friend and showing respect for other people. When the contract is signed the student receives a membership card. This provides benefits such as activities and discounts sponsored by local businesses, to reinforce positive behaviors. The members may choose to prolong their membership by signing a new contract for one year at a time. In the event of breach of contract the members’ parents/guardians are contacted to discuss the matter. The member can be suspended from the program for a period ranging from one month to the rest of the contract period, but is always welcome to return afterwards.

### Study procedure and context

In the spring of 2011, a plan for evaluating the contract strategy was drawn up by SMART, the participating schools, and the research team from Örebro University. The parties agreed that SMART would implement the strategy and keep the school staff informed about the program. The schools’ responsibilities were to have staff implementing the method, to provide class lists of parents’ addresses that would be forwarded to the research team annually, and to set aside time for the annual surveys to allow students to fill them out during school hours. Once per academic year the schools would also report what health promotion and prevention activities had been performed during the school years that the contract-strategy was carried out. The research team was to survey school students, analyze and report these results to the schools and to SMART, and publish them as international scientific articles as well as Swedish-language articles. This study is part of a larger study focusing on “School as a setting for alcohol and drug prevention” within the framework a special venture financed by the Swedish government [48, 49]. The research program includes quasi-experimental longitudinal studies of different prevention programs.

### Study aim and purpose

We aim to evaluate whether the contract strategy, as implemented by the NGO SMART, is successful in preventing youth substance use and problem behaviors. Two research questions are posed: (1) does the signing of contracts have any effect on the levels of substance use? (2) Is the number of years that students have contracts important for the results?

## Methods

The present study design is non-experimental and the intervention was already running prior to the start of our observations. SMART and the schools in this particular municipality had implemented and worked with the strategy since the students who make up this study population were in 4^th^ grade. The research team got involved and conducted the first data collection in autumn 2011 (T1) when students were in 7^th^ grade. Follow-ups were conducted one year later, in autumn 2012, in 8^th^ grade (T2), and in spring 2014, in 9^th^ grade (T3). The survey includes questions about family, school and peer relationships; outlook on life; tobacco, alcohol and drugs; health; and lifestyle. The students answered the questionnaire in the classroom during school hours, in the presence of a representative from the research team who was previously unknown to them.

### Ethics

A letter of consent was sent to parents informing them about the study’s purpose and that participation was voluntary for their children. Parents were given the possibility of providing passive consent; i.e. they only needed to contact us if they did not want their child to participate in the study. Parents were also welcome to contact the research team if they had any questions. Telephone numbers and e-mail addresses were provided. The students received written and verbal information about the purpose of the study. They were also informed that participation was voluntary, about the confidentiality of the data, and that no identifying information would remain accessible. The Ethical Review Board in Uppsala, Dnr. 2011/213, has ethically approved the study, including the opt-out parental consent procedure used.

### Participants

The study population consists of adolescents from five different schools in one medium-sized Swedish municipality. At T1, in 7th grade, students are 13–14 years of age and 50.4 % are boys. At T2, in 8th grade, students are 14–15 years old and 50.6 % are boys. At T3, in 9th grade, the students are 15–16 years and 50.1 % are boys. Response-rates for the survey at the three time-points of data collection are shown in Table 1.

**Table 1:** Total study population and response-rates

	T1	T2	T3
Total-population	*n* = 518	*n* = 502	*n* = 476
Response-rate	*n* = 432	*n* = 458	*n* = 422
	83 %	91 %	89 %

### Measures

#### Sex

Sex was coded 1 for boy and 2 for girl.

#### One non-nordic parent

At least one parent born in a non-Nordic country is coded as 0, and both parents born in Sweden or another Scandinavian country as 1.

#### Monthly allowance

Students were asked how much money (in SEK) they received to spend in their free time and on hobbies on a 7-point scale: (1) “0–249”, (7) “More than 1500”. This is an indicator of SES.

#### Books at home

Students were asked, “How many books are there in your home?” on a 7-point scale, from (1) “No books”, to (7) “More than 500 books”. We did not have information concerning parents’ education and employment; instead we used number of books at home as a socio-cultural indicator [50, 51].

#### Type of residence

The item “Where do you live?” had five response options ranging from (1) “Rental apartment”, to (5) “other accommodation”. Any other type of living than rented counted as owned, and may indicate a higher SES-status. Rented was coded as 1 and owned as 2.

#### Number of years with a contract

Students were asked to report whether they signed a contract for any of the school years ranging from (1) “Yes, in 4^th^ grade”, to (7) “Never signed”.

#### Smoking

Students were asked to report their smoking habits on a 7-point scale: (1) “No, never smoked”, (7) “Yes, every day”. Higher values indicate more established smoking behavior.

#### Snus use

Snus is a form of moist snuff common in Sweden. Students were asked to report whether they use snus and how often on a 7-point scale: (1) “No, never used snus”, (7) “Yes, every day”.

#### Alcohol use

Students were asked to report their alcohol use on a 4-point scale, ranging from (1) “Have not consumed alcohol” to (4) “Have drunk several times”. Higher values indicate higher levels of alcohol use.

#### Drunkenness

Adolescents were asked to report whether they had ever become drunk on a 6-point scale, ranging from (1) “I have never drunk alcohol” to (6) “Yes, every time I drink alcohol I get drunk”. Higher values indicate more frequent binge drinking.

#### Delinquency

Students were asked to report on two items, how many times they had stolen something or intentionally damaged something in the past year on a 5-point scale (two items: *r* = .47 at T1, .47 at T2, .43 at T3): (1) “Never done it” to (5) “More than 10 times”. These two items combined make up a construct representing delinquent tendencies. This is also the only measurement that includes two items, hence the *r* value. Higher scores indicate higher delinquency levels.

#### Bullying

Students were asked how often they been involved in bullying other students in school this semester on a five-point scale from: (1) “I have not bullied anyone at school this semester”, to (5) “Several times a week”. The following written definition of bullying was provided in the survey: A student is bullied when another student (or group of students) says or does nasty and unpleasant things to him or her. It is also bullying when a student is constantly teased in a way he or she does not like. It is not bullying when two fairly evenly matched students quarrel or fight. It is also not bullying when a student teases another student in a kind or friendly manner.

### Data analysis

The IBM SPSS software package version 22 was used for statistical analysis. Using the data at T3 on the number of years with a contract, students were grouped into three groups in terms of how long they have participated in the program: students who had a signed contract for 5 years, from 4^th^ until 7^th^ grade plus 8^th^ and/or 9^th^ grade (long-term participants, 22.4 %), students who signed a contract in 4^th^ grade and continued for the coming 2–4 years (short-term participants, 39.9 %), and students who never signed a contract or only had a signed contract in some non-consecutive years (0–3 years) (sporadic- or non-participants, 20.6 %). This operation turned the analytic sample into one with a total of 414 participants. Creating a comparison condition using non-participants has previously been used successfully [52] and is accepted as a reasonable strategy for increasing the internal validity of a study’s conclusions [53]. The group of sporadic- or non-participants served as the comparison condition in our analyses.

Our main analysis used GLM repeated measures ANOVA to test if the outcome measures smoking, snus and alcohol use, level of drunkenness, delinquency and bullying changed significantly over time differently for the different groups. We then used a one-way ANOVA to compare long-term, short-term, and sporadic- or non- (SPON) participants in the program on demographic background variables, and all the outcome measures mentioned above assessed at all measurement occasions. In order to reach more robust conclusions regarding the magnitude of group differences at the end of the study we estimated effect-sizes according to Cohen’s *d* [54]. Furthermore, we used ANCOVA to compare the groups on the outcomes controlling for sex and the SES variable books at home, as preliminary results showed significant baseline differences between groups for these specific variables. This would partial out the initial differences between groups, so that the differences in outcomes at measurement occasions may be attributable to number of years with a contract. The variables were analyzed as ratio scale variables; we considered them as such on the basis of the many possible answers on the questionnaire [55]. All results were considered significant at *p* ≤ 0.05.

In order to analyze and understand the missing data pattern we first recoded the six outcome variables, with 1 for missing cases and 0 for everything else. In this way we could inspect the frequencies of missing cases in the main study variables. This indicated internal missingness – a consistent level of missingness across all the variables and time-points: 15–17 % missing at T1 on all six outcome variables, around 10 % missing at T2, and 15–17 % missing at T3. A one-way ANOVA was then conducted in which all the recoded variables were run by the categorical variable number of years with a contract. In this way we could identify differences in missingness at each measurement occasion between the groups. The short-term and SPON-participants were missing the most data on smoking at T1 and T2. No other significant differences in missingness were found.

## Results

All outcomes significantly increased over time for the whole sample (Table 2). Initial results showed that students who participated in the program for the longest time, and signed contracts for at least 5 years from 4^th^ grade on, had significantly lower levels of youth problems at T3 than students in the other two groups. However, further inspection of the data revealed a significant difference in sex distribution between the three groups. There are more boys (67 %) in the group of SPON-participants than there are girls. There is also a significant difference between the groups on the SES variable books at home. Students in the group of long-term participants report having more books at home (M = .70) than students in the group of short-term participants (M = .62), and SPON-participants (M = .52). These variables are controlled for in ANCOVA analyses.

**Table 2:** Means and standard deviations, and differences between groups on study variables

	Long-term participants		Short-term participants		SPON-participants			
	(*n* = 112)		(*n* = 200)		(*n* = 102)			
	M	SD	M	SD	M	SD	F-test	*p*
Baseline demographic and SES variables								
Sex	1.59^x^	.49	1.51^x^	.50	1.33^z^	.47	7.14 (2,357)	.001
One non-Nordic parent	.82	.38	.88	.33	.77	.42	2.37 (2,352)	.095
Monthly allowance	.19	.23	.24	.26	.18	.24	1.69 (2,353)	.186
Books at home	.70^x^	.24	.62^y^	.23	.52^z^	.26	13.10 (2,354)	.001
Type of residence	1.06	.24	1.07	.26	1.08	.28	.194 (2,357)	.824
Smoking								
T1	.12	.78	.19	.60	.19	.53	.35 (2,354)	.704
T2	.16	.79	.36	.94	.47	.99	2.94 (2,380)	.054
T3	.28^x^	.71	.84^x^	1.47	1.12^z^	1.71	10.64 (2,411)	.001
Snus use								
T1	.07	.60	.06	.42	.08	.31	.04 (2,357)	.961
T2	.07	.62	.13	.58	.26	.79	2,00 (2,379)	.137
T3	.16^x^	.55	.59^x^	1.41	.73^z^	1.33	6.75 (2,408)	.001
Alcohol use								
T1	1.47	.69	1.64	.77	1.73	.86	2.96 (2,353)	.053
T2	1.57^x^	.77	1.98^x^	1.01	1.91^z^	1.00	6.56 (2,379)	.002
T3	2.04^x^	1.06	2.57^x^	1.21	2.59^z^	1.13	8.65 (2,410)	.001
Drunkenness								
T1	1.34^x^	.78	1.48^xz^	.61	1.60^z^	.82	3.06 (2,353)	.048
T2	1.44	.88	1.60	.90	1.67	1.06	1.64 (2,381)	.195
T3	1.72^x^	1.03	2.43^x^	1.60	2.41^z^	1.53	9.52 (2,408)	.001
Delinquency								
T1	2.14	.86	2.25	.83	2.40	1.11	1.87 (2,352)	.156
T2	2.24	1.00	2.37	1.11	2.47	.86	1.22 (2,380)	.295
T3	2.23^x^	.72	2.52^xz^	1.33	2.76^z^	1.62	4.59 (2,410)	.011
Bullying								
T1	1.1	.58	1.08	.32	.108	.31	.14 (2,356)	.868
T2	1.05	.23	1.14	.49	1.16	.45	1.80 (2,379)	.167
T3	1.06	.41	1.11	.46	1.16	.48	1.17 (2,409)	.313

Groups with different superscript letters did differ (*p* < 0.05) on measured outcomes (ANOVA, Tukey’s HSD), and groups with similar superscripts did not significantly differ

### Smoking

**Fig. 1:**
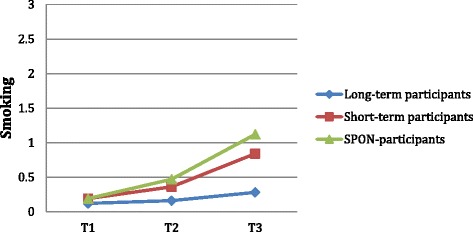
Changes in levels of self-reported smoking behavior

The difference between groups in smoking at T3 remained significant after being adjusted, suggesting that it may be attributed to the number of years with contract. We also see a significant difference in how smoking increased in different groups over time, *F*(4,656) = 4.69, *p* = .001 (see Fig. 1). At baseline (T1), the groups are alike in self-reported smoking behavior. Differences between the groups start approaching significance at T2 (*p* = .054), and are significant at T3 (*p* = .001). The effect size between long-term participants and short-term participants at T3 is nearly medium (*d* = .48). Between long-term participants and SPON-participants the effect size at T3 is medium (*d* = .64), and between short-term participants and SPON-participants the effect size at T3 is small (*d* = .18).

### Snus use

The significant difference in snus use between groups at T3 is also explained by sex, *F*(1,325) = 20.01, *p* = .001, and can thus not solely be attributed to the number of years with contract. We see a significant difference in how snus use increased in different groups over time, *F*(4,652) = 4.53, *p* = .001. There are significant differences between the groups only at T3 (*p* = .001). At that time point long-term participants had the lowest reported snus use (M = .16), compared to short-term (M = .59) and SPON-participants (M = .73). The effect size between long- and short-term participants at T3 is small to moderate (*d* = .40), between long-term and SPON-participants it is medium (*d* = .56), and between short-term and SPON-participants it is small (*d* = .10).

### Alcohol use

**Fig. 2:**
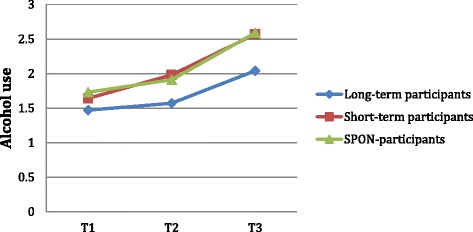
Changes in levels of self-reported alcohol use

The difference in alcohol use between groups at T3 remained significant after being adjusted, suggesting it may be attributed to the number of years with contract. We see a significant difference between the groups in self-reported alcohol use starting at T2 (*p* = .002). Long-term participants have the lowest levels of alcohol use (M = 1.57). Interestingly, short-term participants have the highest levels of alcohol use at T2 (M = 1.98), and SPON-participants are slightly below them (M = 1.91). This difference between the groups in alcohol use at T2 remained significant after being adjusted. At T3, long-term participants have increased their level of alcohol use (M = 2.04) but it is still significantly lower than that of short-term (M = 2.57) and SPON-participants (M = 2.59). The rate of change from T1 to T3 in alcohol use is significantly different between groups, *F*(4,450) = 2.50, *p* = .041 (see Fig. 2). The effect size between long-term participants and short-term participants in alcohol use at T3 is nearly medium (*d* = .47), and that between long-term and SPON-participants is medium (*d* = .50). Between short-term and SPON-participants this difference is almost non-existent at T3 (*d* = .01).

### Drunkenness

**Fig. 3:**
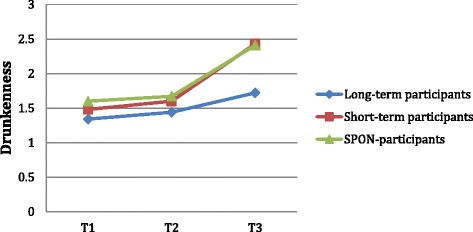
Changes in self-reported drunkenness

The difference between groups at T3 in self-reported drunkenness also remained significant after being adjusted; again, suggesting it may be attributed to the number of years with contract. Drunkenness is the only outcome measure where we see a significant difference between groups already at T1 (*p* = .048). Long-term participants, who at this time-point already had been signing yearly contracts for three years (since 4^th^ grade), report significantly lower levels of drunkenness (M = 1.34) than short-term participants (M = 1.48) and SPON-participants (M = 1.60). However, the variable books at home also explain the variance in the difference between the groups in drunkenness at T1, *F*(1,323) = 6.99, *p* = .009. At T2, the difference in drunkenness between the groups is no longer significant, but it regains significance at T3. Drunkenness levels increased more sharply for the groups of short-term and SPON-participants from T2 to T3 (see Fig. 3). Despite an inconsistent pattern over time, we see a significant difference overall in how levels of reported drunkenness increased in different groups from T1 to T3, *F*(4,652) = 6.28, *p* = .001. The effect size between long-term and short-term participants for drunkenness at T3 is medium (*d* = .53), as is that between long-term and SPON-participants (*d* = .54). Between short-term and SPON-participants it is small (*d* = .01).

### Delinquency

The significant difference between groups in delinquency at T3 is also explained by sex, *F*(1,323) =11.11, *p* = .004, and can thus not fully and solely be attributed to the number of years with contract. We see a non-significant difference in how groups changed over time from T1 to T3 on indicators of delinquency, *F*(4,648) = .33, *p* = 868. Between long- and short-term participants the difference in reported delinquent acts at T3 is significant (*p* = .001) but small (*d* = .27), as is that between short-term and SPON-participants (*d* = .16). Between long-term and SPON-participants the difference in reported delinquency at T3 is however moderate (*d* = .43).

### Bullying

Results revealed a non-significant difference between the groups in the outcome bullying. As shown in Table 2, all three groups have almost the lowest values possible in this measure at all measurement occasions.

## Discussion

The present study was conducted as part of an evaluation of the contract strategy as implemented and conducted by an NGO, and contributes to the development of an evidence base related to school-based alcohol and tobacco interventions. One goal of the study was to obtain information on whether writing a contract has an inhibiting effect on levels of substance use among schoolchildren. The findings in this study indicate that the longer that students take part in the SMART program and have a signed contract, the less they smoke, use snus, drink alcohol, get drunk, and commit delinquent acts when they are in 9^th^ grade. Above all, the present study addresses the need for scientific evaluation of methods focusing on primary prevention in schools in the Swedish context.

As a complement to the existing Swedish education on alcohol, narcotics, doping, and tobacco in schools, which already is interactive [56], the SMART contract strategy is a multicomponent intervention using positive reinforcement and involving the adolescents’ social environment, parents and community. The component of requiring a parent to give written consent leads to conversations at home on the contract items [57]. Furthermore, the SMART contract strategy addresses norms, as well as the intention and commitment not to use substances. All of the above are components that previous research has identified as effective in prevention programs [28–32, 37], they target determining factors of smoking and alcohol use in adolescence previously identified [17–26], and the strategy has a further strong advantage in its flexibility and adaptability to local conditions.

Regarding the study findings, controlling for sex and SES, we can infer program effect to some extent because the rate of change over time in outcomes amongst students in the short-term and SPON groups was significantly higher than that in the long-term group. The time effect – that a significant observable overall increase in, for instance, youth smoking or alcohol use, as our measures are constructed (i.e. “have taken a sip from someone else’s glass”), occurs from 7^th^ to 9^th^ grade – is more or less expected to occur in all groups and is therefore less surprising.

Another matter that should be emphasized regarding the findings is that the groups of individuals who participated in the program for fewer years and scored significantly higher on the surveys than the group of individuals who participated in the program for more years still do not report greatly increasing levels of substance use relative to our measurement scale. Indeed, there are statistically significant differences between them. However on the 7-point scale that we, for example, used to measured smoking, none of the groups scored above 1.71. So as a group, on the question “Do you smoke”, the SPON-participants, who are the highest-scoring group, approach “No, but I have tried it” in 9^th^ grade. In regard to drunkenness, the two highest scoring groups (short-term and SPON-participants) scored only 2.43 and 2.41 out of possible 6 points, respectively, meaning that they had drunk alcohol to the limit of intoxication only once, up to that time point in 9^th^ grade. This is not to trivialize youth drunkenness, but when considering the practical difference between the groups and the practical significance of the finding, it is fairly low. This is of course a good thing, and may be explained by the prevailing culture and other established approaches among the schools in the particular municipality, especially because at least two of the schools provided other health promotion and prevention activities in addition to the ordinary curriculum and the SMART program. It is most certainly also a result of more comprehensive national and regional strategies that have achieved a population-level impact [58]. It has been suggested that the latter are necessary because school-based alcohol, tobacco and drug prevention programs alone generally have small effects [37, 59]. Nevertheless, on a group level, the present study shows a solid medium-sized difference between having been involved in the SMART program for at least 5 years starting in 4^th^ grade and not having done so, when looking at drunkenness.

It has been suggested in previous studies that primary prevention programs – particularly for reducing alcohol [60] and tobacco use [19] among adolescents – should be provided before 6^th^ grade, or at least before initiation occurs. That is in order to influence views and attitudes regarding substances before adolescents come across them elsewhere, as they usually do in the upper grades of compulsory school. The present study adds further support to these ideas.

Our findings also suggest the necessity of prolonging the period of participation in the program. Looking at the differences in alcohol use and levels of drunkenness between the group of short-term participants and SPON-participants in 8^th^ and 9^th^ grade, we see they are small, and not even statistically significant. This indicates that students who quit the program, as short-term participants do, do not opt out of substance use to the same extent as those continuing attending the program year after year, and as a result their levels of substance use were rising. Besides finding this annual signing of a new contract for the coming year as a possible booster session [61], the aim of this paper is not to provide any strategic plan for how to accomplish continued participation in signing contracts.

The non-significant differences between the groups for the effect of the program on levels of bullying is a result in itself, but can also partly be understood as a methodological technical issue. Even though the participants reported scores that were almost the lowest possible, the one-item measure we used is probably not adequate. In the bullying and peer-victimization research literature we find more comprehensive assessment tools with more nuanced scales able to measure this more accurately [62, 63]. Also, the low levels of bullying detected even by our measure may be due to the pre-existing interdisciplinary anti-bullying plans explicitly described by representatives of at least two of the schools in an interview with a member of the research team.

One final aspect worth highlighting is that the SMART contract-signing strategy has no built in control system. We lack information on whether every possible breach of contract was detected and handled, especially as it most likely would have occurred outside school-hours. However, regarding the validity of self-reports of socially unacceptable behaviors such as adolescent smoking and alcohol drinking, a cross-sectional, biochemically verified analysis of a Swedish cohort sub-sample confirmed that adolescents’ self-reported tobacco use is reliable [64]. Regardless of this reliability, it is possible that someone signed a contract but violated it while enjoying the benefits and reporting false answers in the survey. The SMART contract-strategy largely relies on the individual’s own conscience and sense of responsibility.

### Limitations and strengths

One limitation of the study is that the evaluation was carried out in five schools within one Swedish municipality, as the SMART interventions are tailor-made by local agents to conform to the needs and wishes of the locale within which they are implemented. Thus, the results of this evaluation might not be easily generalized to SMART programs in other locales. Another point worth noting regards the measures we use. These are very limited and far from exhaustive, and should be considered approximations. To use non-ratio items as ratio-scales can be seen as a limitation. Moreover, the collection and documentation of information on schools’ health promotion and prevention activities, which originally was to be collected and documented each academic year during the study, was only done for one year during the study. Yet a concern in the present study is that the group differences in missingness on one variable were not further analyzed, which may have introduced potential bias and affected the results. No adjustments were made for multiple comparisons. Neither can we claim robust evidence for program effect due to evaluation design. On the other hand, if individuals with greater exposure to the program show greater change in the outcomes, it strengthens the argument that the program led to changes. In our study this exposure would consist of annually signing the contract, stating one’s intention not to use substances, and actively deciding to opt out of unwanted behaviors. The study’s limitation to generalize the results to other locales includes also a strength of the SMART strategy, namely the possibility to adapt a method locally. A further strength of the study is the sample size, providing adequate statistical power that makes it possible to detect effect sizes [54]. We also chose not to have categorical or dichotomous outcome variables as there is a risk of losing one to two thirds of the information on the variance of the total sample [65]. The high participant response-rate and low missingness of the outcome measures further strengthens our conclusions.

### Directions for future research

While the SMART contract strategy consists of evidence-based components and the present study provides support for its effectiveness, there are still questions to be answered. The individuals who chose to take part in the program and sign contracts for several years might have refrained from smoking and drinking even without the contract. We need to know the characteristics of individuals who choose to sign a contract every year. Predictors for reporting having had a contract during all the years need to be further analyzed and identified. More importantly would be to explore what characterizes individuals who decline to participate in the program. This could guide the development of a strategic plan for how to accomplish continuing participation in the program and how to reach non-participants.

## Conclusion

Our findings show that individuals who were less exposed to and involved with the SMART contract program developed significantly higher levels of substance use during the course of the study than those with more exposure to the program. This suggests that the SMART program has some preventive effects on adolescent substance use. The current findings are not conclusive however, and future research is needed to reach more robust conclusions about the effectiveness of the SMART contract strategy. Implications of these findings for practice are that continued support should be given to actors working with this form of contract-activity, and that efforts should focus on getting more students to sign contracts for several years. Implementation and development of the program should take into account and be based on the local context.

## Abbreviations

ANCOVA, analysis of Covariance; ANOVA, analysis of variance; GLM, general linear model; NGO, non-governmental organization; SES, socioeconomic status; SFC, smoke-free class competition; SPON participants, sporadic- or non-participants.
